# Urbanicity and Paediatric Bacteraemia in Ghana—A Case-Control Study within a Rural-Urban Transition Zone

**DOI:** 10.1371/journal.pone.0139433

**Published:** 2015-09-29

**Authors:** Peter Sothmann, Ralf Krumkamp, Benno Kreuels, Nimako Sarpong, Clemens Frank, Lutz Ehlkes, Julius Fobil, Kennedy Gyau, Anna Jaeger, Benedicta Bosu, Florian Marks, Ellis Owusu-Dabo, Bernd Salzberger, Jürgen May

**Affiliations:** 1 Research Group Infectious Disease Epidemiology, Bernhard Nocht Institute for Tropical Medicine, Hamburg, Germany; 2 Division of Tropical Medicine, 1st Department of Medicine, University Medical Center Hamburg-Eppendorf (UKE), Hamburg, Germany; 3 German Centre for Infection Research (DZIF), partner site Hamburg-Borstel-Lübeck, Hamburg, Germany; 4 Kumasi Centre for Collaborative Research in Tropical Medicine, Kumasi, Ghana; 5 Institute of Microbiology and Hygiene, Charité-University Medicine, Berlin, Germany; 6 School of Public Health, University of Ghana, Accra, Ghana; 7 International Vaccine Institute (IVI), Seoul, Korea; 8 Department of Internal Medicine I, University Hospital Regensburg, Regensburg, Germany; University College London, UNITED KINGDOM

## Abstract

**Background:**

Systemic bacterial infections are a major cause of paediatric febrile illness in sub-Saharan Africa. Aim of this study was to assess the effects of social and geographical determinants on the risk of bacteraemia in a rural-urban transition zone in Ghana.

**Methods:**

Children below 15 years of age with fever were recruited at an outpatient department in the suburban belt of Kumasi, Ghana’s second largest city. Blood was taken for bacterial culture and malaria diagnostics. The socio-economic status of participants was calculated using Principle Component Analysis. A scale, based on key urban characteristics, was established to quantify urbanicity for all communities in the hospital catchment area. A case-control analysis was conducted, where children with and without bacteraemia were cases and controls, respectively.

**Results:**

Bacteraemia was detected in 72 (3.1%) of 2,306 hospital visits. Non-typhoidal *Salmonella* (NTS; n = 24; 33.3%) and *Salmonella typhi* (n = 18; 25.0%) were the most common isolates. Logistic regression analysis showed that bacteraemia was negatively associated with urbanicity (odds ratio [OR] = 0.8; 95% confidence interval [CI]: 0.7–1.0) and socio-economic status (OR = 0.8; 95% CI: 0.6–0.9). Both associations were stronger if only NTS infections were used as cases (OR = 0.5; 95% CI: 0.3–0.8 and OR = 0.6; 95% CI: 0.4–1.0, respectively).

**Conclusions:**

The results of this study highlight the importance of individual as well as community factors as independent risk factors for invasive bacterial infection (IBI) and especially NTS. Epidemiological data support physicians, public health experts and policy makers to identify disease prevention and treatment needs in order to secure public health in the transitional societies of developing countries.

## Introduction

In 2012, three out of four deaths in African children under the age of five were due to communicable diseases and acute febrile illness was the most common cause of hospital admission on the continent [1,2]. Malaria is the predominant cause of systemic febrile illnesses in sub-Saharan Africa. Yet, additional causes of fever are increasingly the focus of research and public health campaigns [3,4]. Studies on paediatric febrile illness emphasize the role of invasive bacterial infections (IBI), which were found in up to 13% of febrile hospital admissions [1]. Moreover, little is known about the social and geographical determinants of IBI in Africa [5–7]. So far, only two studies from Kenya have described rural/urban differences in the incidence of *Salmonella* infections. Non-typhoidal *Salmonella* (NTS) was the leading rural pathogen with an incidence rate ten-fold higher compared to an urban site [6]. *S*. *typhi*, in contrast, had a fifteen-fold higher incidence rate amongst the urban population [7]. These findings suggest that factors related to the level of urbanicity may affect the distribution of IBIs. However, it is unclear whether individual or community factors are the major determinants of this discrepancy.

Developing countries are in the midst of massive social and economic transition. It is estimated that by 2050 Africa’s urban population will have increased from 400 million to 1.2 billion. As a result, informal settlements with poor levels of infrastructure will extend and social differences will increase [8]. These profound changes in lifestyle and living conditions are likely to affect infectious disease epidemiology [9]. A better understanding of the epidemiological impact of social transition is crucial for future public health strategies in developing countries.

The aim of this study was to assess the effects of social and geographical determinants on the risk of bacteraemia in a rural-urban transition zone in Ghana. To account for the complexity of urbanization processes in developing countries [10], an urbanicity scale was developed, using rural/urban-key features representing the various aspects of urbanization in a developing region. In addition, the individual socio-economic status of study participants was estimated and both community-level and individual effects on the risk of childhood bacteraemia were assessed.

## Methods

### Study area

The study was conducted in the suburban belt of Kumasi, the capital of the Ashanti Region in Ghana. Kumasi is the region’s economic and administrative centre and, with a total population of two million people, Ghana’s second largest city. An annual population growth rate of 7.4% over the last decade has led to the expansion of Kumasi’s periurban zone, particularly along the principal exit roads [11]. Study participants were recruited at St. Michael’s Hospital (SMH) in Pramso, a town with 3,259 inhabitants located 20 km southeast of Kumasi along one of the major roads leaving the city. SMH is the biggest health care facility in the Bosomtwe District with a catchment area covering both Kumasi Metropolis and the Bosomtwe District. The Bosomtwe District stretches from the suburbs of Kumasi to a largely rural countryside. Most of the primary vegetation has been cleared, giving way to predominantly bush and farmlands. Malaria is endemic with high transmission rates throughout the year [12]. Ghana’s under-five mortality rate is 72 deaths per 1000 live births and the HIV prevalence is 1.4% amongst the adult population [13].

The Committee on Human Research, Publications and Ethics at the School of Medical Sciences, Kwame Nkrumah University of Science and Technology in Kumasi approved the study protocol and informed consent process.

### Study population

Between January and December 2012, all children below 15 years presenting to the Outpatient Department (OPD) at SMH with fever (tympanic temperature of ≥38°C) were recruited if their caregiver gave written informed consent. Medical history and further socio-demographic data were obtained.

### Laboratory diagnostics

A blood sample was taken from every child by venepuncture for malaria diagnosis and bacterial blood culture. One to three millilitres of blood were injected into vials for paediatric blood cultures (Becton Dickinson, NJ 07417, USA) and incubated in an automated BACTEC 9050 culturing instrument (Becton Dickinson). Broth from positive bottles was examined microscopically (Gram stain) and was further cultured on standard media (chocolate agar, MacConkey agar, and Columbia agar with 5% sheep blood). For malaria diagnosis Giemsa-stained thin and thick blood slides were prepared. Two independent readers examined the slides and a third reading was performed in case of discrepancies. In 39 (1.7%) patients malaria results were not available, and these patients were excluded from the respective analyses.

### Urbanicity scale

A continuous numeric scale of urbanicity was constructed and validated according to the principles of scale development as described by DeVellis [14] and Netemeyer & Bearden [15]. To construct the scale, data on community characteristics were selected by literature review as well as extracted from the “2010 Population & Housing Census” conducted by the Ghana Statistical Service [16]. Additional information on available private services, road conditions and public transport within the communities was collected via systematic interviews in the communities. Variables were grouped into eight thematic scale components; namely population size, economic activity, education, health services, transportation, public and private services, sanitation, and housing. Each of these components accounted for ten points while variables within a component were weighted equally. Consequently, the final scale ranged from 0 to 80 points. To obtain a score for each community, points were accumulated according to the community’s particular characteristics. Unidimensionality of the scale was tested by exploratory factor analysis (EFA), Kaiser-Meyer-Olkin (KMO) measure was used to assess sampling adequacy and internal consistency was validated using Cronbach’s alpha [17]. For more details on scale construction and validation results please refer to the supplementary material (Supplement 1).

### Socio-economic status

To estimate the socio-economic status (SES) at an individual level, a scale was established using Principal Component Analysis (PCA) [18]. It was conducted with variables containing individual data on mother’s education, living conditions (i.e., type of cooking energy, source of drinking water, type of sanitation) and ownership of assets (i.e., mobile phone, television, computer, fridge). The model’s correlation matrix was constructed using tetrachoric correlation coefficients for dichotomous variables. Again, sampling adequacy and internal consistency were assessed by KMO measure and Cronbach’s alpha, respectively.

### Statistical analysis

Participants with a positive blood culture served as cases, while controls had a negative blood culture result. A second case-control set up was done on isolate level, where NTS infection, the most common blood isolate in the study, defined cases while the same control group was used. Patients who showed growth of probable or possible contaminants (e.g., *Bacillus spp*., *Micrococcus spp*. or coagulase-negative *Staphylococcus spp*.) were excluded from the study.

Associations with bacteraemia or NTS infection were calculated by logistic regression and displayed as odds ratios (ORs) with their corresponding 95% confidence intervals (CI). For this purpose, SES and urbanicity scores were categorized into four quartile-based groups (i.e., first quartile contains lowest SES/urbanicity score; fourth quartile contains highest SES/urbanicity score). Spearman’s Rank Correlation Coefficient was calculated to assess associations between categorical variables. Finally, multivariate logistic regression models were established to calculate adjusted odds ratios (aOR) accounting for potential confounding. All analyses were carried out using Stata 12 (StataCorp LP, College Station, USA).

## Results

A total of 2,306 hospital visits were included in the analysis. Patients were hospitalized for 375 (16.3%) of these visits, with an average hospital stay of 2.8 days [standard deviation (SD): ±1.3]. Girls were slightly underrepresented in the study group (n = 1,071; 46.4%). The median age of the study children was 34 months (Interquartile range [IQR]: 16–63 months). Characteristics of the study participants are summarised in Table 1.

**Table 1:** Characteristics of cases and control living in the rural/urban study area.

Characteristics	Cases	Controls
Total, n (%)	72 (3.1)	2,234 (96.9)
Median age, months (IQR)	30 (18–65)	35 (16–62)
Female, n (%)	30 (41.7)	1,041 (46.6)
Malaria, n (%)	16 (22.2)	850 (38.8)
Median urbanicity, score (IQR)	46.5 (27.3–54.0)	53.5 (35.3–58.3)
Urbanicity quartile 1, n (%)	29 (40.3)	547 (24.5)
Urbanicity quartile 2, n (%)	13 (18.1)	440 (19.7)
Urbanicity quartile 3, n (%)	14 (19.4)	512 (22.9)
Urbanicity quartile 4, n (%)	16 (22.2)	735 (32.9)
Median SES, score (IQR)	1.4 (0.9–1.9)	1.7 (1.1–2.2)
SES quartile 1, n (%)	24 (33.3)	527 (23.6)
SES quartile 2, n (%)	24 (33.3)	573 (25.7)
SES quartile 3, n (%)	16 (22.2)	537 (24.0)
SES quartile 4, n (%)	8 (11.1)	597 (26.7)

Abbreviations: SES, socio-economic status; IQR, interquartile range.

### Laboratory results

Bacteraemia was detected in 72 (3.1%) patients, who served as study cases. Median age of cases was 30 months (IQR: 18–65). Most frequent pathogens were NTS (n = 24; 33.3%), *Salmonella* Typhi (n = 18; 25.0%) and *Streptococcus pneumoniae* (n = 15; 20.8%). Controls, which were blood culture negative, had a median age of 35 months (IQR: 16–62).

Malaria parasites were detected in 16 (22.2%) cases and 850 (38.8%) controls. In 1,345 (61.3%) patients neither bacteraemia nor parasitaemia were detected.

### Study villages and urbanicity

**Fig 1:**
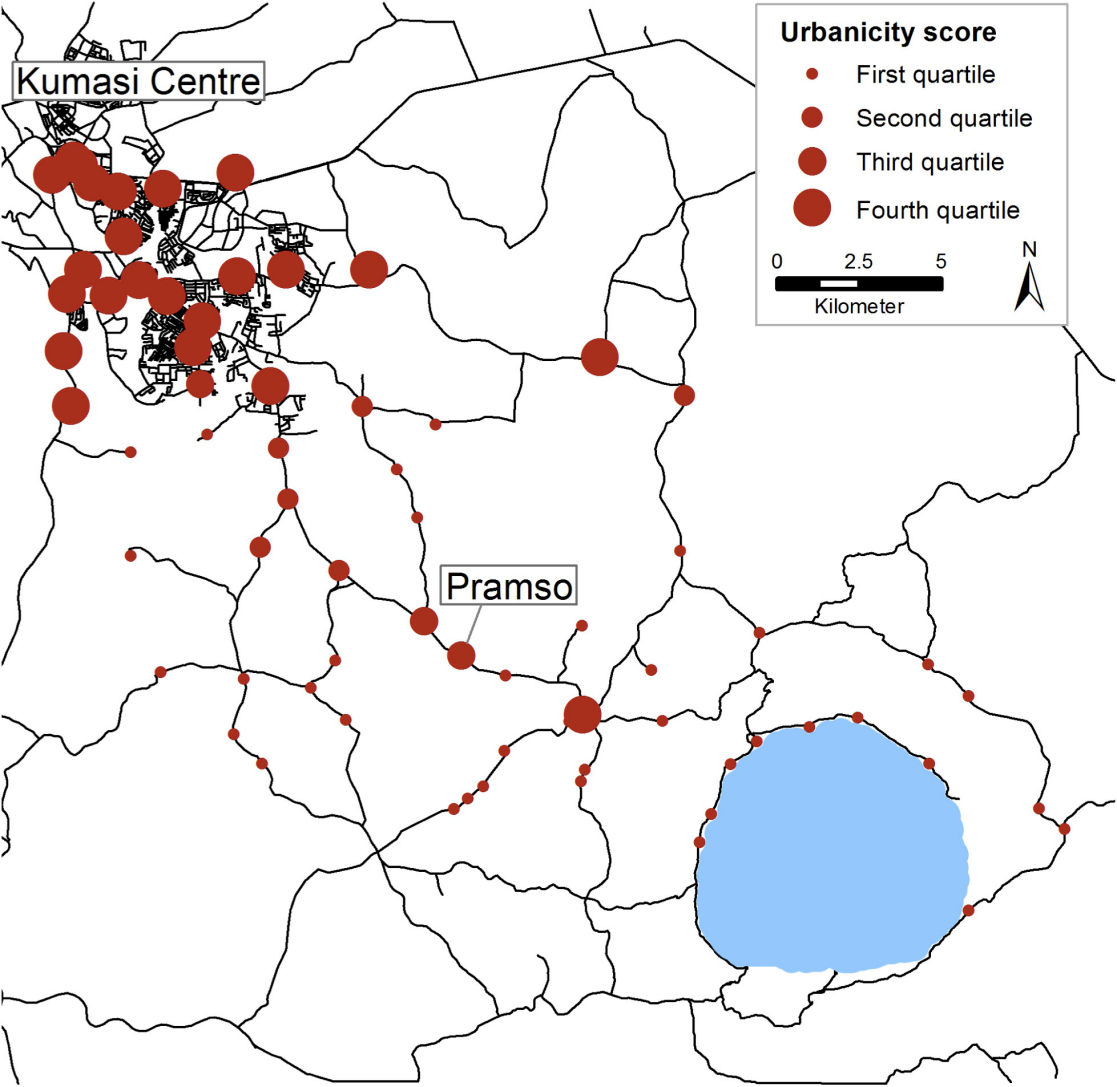
Map of the study area located within the Ashanti Region, Ghana. The level of urbanicity of a community is indicated by the size of the red dots.

The catchment area of SMH covered 73 communities spread over 375 km^2^. Forty-nine (67.1%) communities were located in Bosomtwe District, 22 (30.1%) in Kumasi Metropolis and one each in Ejisu Juaben District and in Atwima Kwanwoma District. The population size per community ranged from 214 to 72,105 inhabitants, with a median of 1,608 (IQR: 738–8,150) inhabitants. The communities scored a minimum of 6.0 and a maximum of 78.5 points on the urbanicity scale, with a median of 31.3 (IQR: 20.5–65.3) points. A map with the study villages and their level of urbanicity is shown in Fig 1.

Validation of the scale showed good unidimensionality (eigenvalue of the first factor = 6.1; overall variance explained by the first factor: 96.6%), good sampling adequacy (KMO measure for the first factor = 0.93), and a high internal consistency (Cronbach’s alpha = 0.96).

### Socio-economic status

To quantify the individual SES of study participants, a PCA was conducted on eight socio-economic characteristics. The analysis yielded an eigenvalue of 4.1 for the first component, with eigenvalues of 1.0 and below for the remaining components. A SES score was calculated from the first component of the PCA, which accounted for an overall variance of >50%. The KMO measure of 0.86 indicated good sampling adequacy, KMO values for all used variables were greater than 0.78. Cronbach’s alpha of 0.70 showed moderate internal consistency.

### Urbanicity, SES and bacteraemia

**Fig 2:**
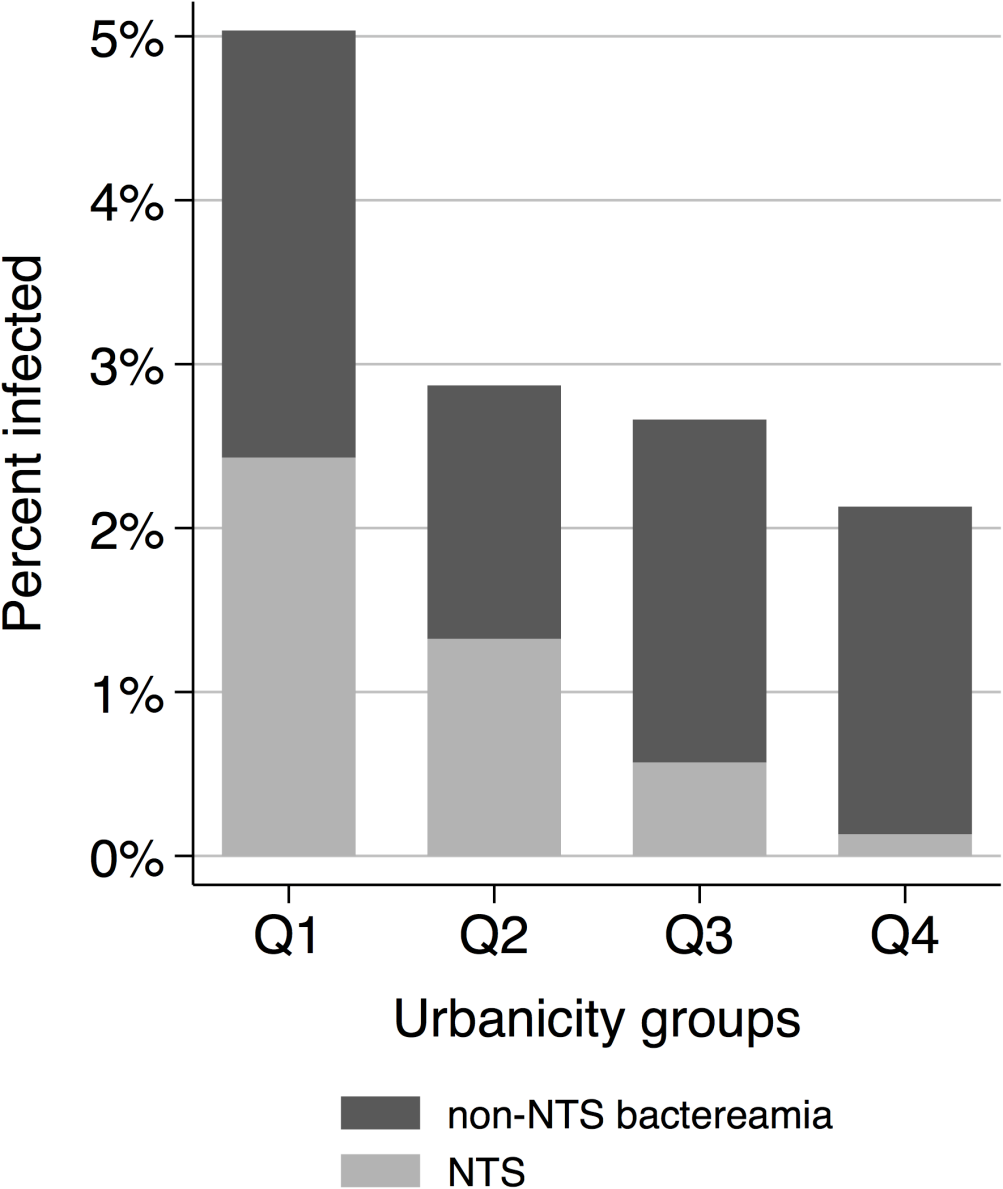
Frequency of non-typhoid *Salmonella* and other bloodstream infections among the four urbanicty groups (Q1 = low urbanicity and Q4 = high urbanicity).

Overall, the proportion of positive blood cultures decreased with increasing urbanicity. From the lowest to the highest urbanicity group bacteraemia was diagnosed in 29 (5.0%), 13 (2.9%), 14 (2.7%), and 16 (2.1%) patients, respectively (Fig 2). In the bivariate analysis, the proportion of patients with bacteraemia decreased with an OR of 0.7 (95% CI: 0.6–0.9) along the urbanicity groups.

Looking at NTS alone, the frequency and proportion of cases showed a marked linear trend along the urbanicity groups. NTS was found in 14 (2.5%), 6 (1.4%), 3 (0.6%), and 1 (0.1%) patients from the lowest to the highest category (Fig 1). The association between urbanicity and NTS was strong with an OR of 0.5 (95% CI: 0.3–0.7) per category step. When NTS cases were excluded from the group of bacteraemia cases no statistical association between bacteraemia and urbanicity could be shown (OR = 0.9; 95% CI: 0.7–1.2).

**Fig 3:**
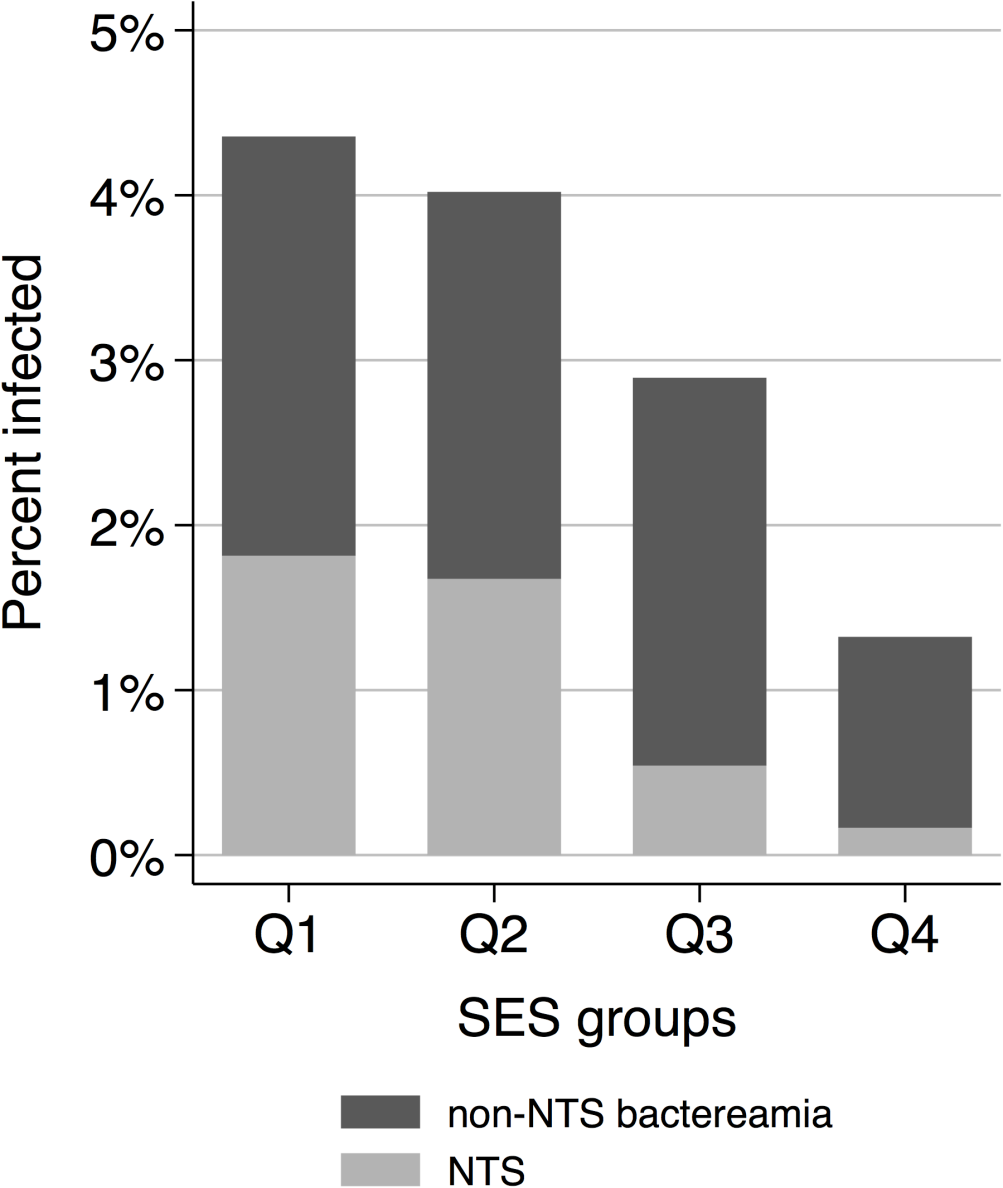
Frequency of non-typhoid *Salmonella* and other bloodstream infections among the four socio-economic status (SES) groups (Q1 = low SES and Q4 = high SES).

The proportion of patients with bacteraemia decreased towards higher SES groups as well. From the lowest to the highest SES group, bacteraemia was found in 24 (4.4%), 24 (4.0%), 16 (2.9%), and 8 (1.3%) patients, respectively (Fig 3). The proportion of patients with bacteraemia decreased with an OR of 0.7 per SES category (95% CI: 0.6–0.9). Among the SES groups, NTS infection was found in 10 (1.9%), 10 (1.7%), 3 (0.6%), and 1 (0.2%) patients (Fig 2). Similarly to urbanicity, the association between SES and NTS was strong (ORs = 0.6; 95% CI: 0.4–0.8), while SES showed reduced associations with bacteraemia when NTS cases were excluded from the analysis (OR = 0.8; 95% CI: 0.6–1.0).

There was evidence for a weak correlation between urbanicity and SES (Spearman’s rho = 0.34; p<0.001), thus multivariate logistic regression models were established to account for potential confounding. In the adjusted models the association of both urbanicity and SES with bacteraemia remained comparable to the crude results (OR = 0.8; 95% CI: 0.7–1.0 and OR = 0.8; 95% CI: 0.6–0.9, respectively). Likewise, comparable effects were observed when modelling the correlation with NTS infection, with ORs of 0.5 (95% CI: 0.3–0.8) and 0.6 (95% CI: 0.4–1.0) for urbanicity and SES, respectively.

## Discussion

Both higher urbanicity and SES were associated with lower odds of systemic bacterial infections in febrile children from a rural-urban transition zone in Ghana. These associations remained when urbanicity and SES were included in multivariate regression models highlighting that both are independent risk factors for IBI. As shown in a previous study [5], associations were mainly driven by NTS, the most frequent isolate in our study sample.

The proportion of bacteraemia decreased from the lowest to the highest urbanicity group. Low urbanicity status is generally associated with increased childhood mortality [19], but so far only a few studies have analysed the association between urbanicity and IBI in sub-Saharan Africa. Biggs et al. showed that the prevalence of bacteraemia was higher in a rural compared to an urban site in Tanzania. These findings were largely driven by NTS, while other pathogens showed no urban and rural differences [5]. In accordance with that, our analyses yielded stronger effects of urbanicity on NTS than on unspecified bacteraemia and urbanicity showed no statistical association when cases of NTS were excluded. A comparable relationship was described from Kenya, where NTS accounted for 39% of rural and only 3% of urban blood culture isolates with an adjusted incidence rate ten times higher in the rural site [6].

We detected bacteraemia in 3.1% of febrile OPD children, a frequency similar to that in other OPD studies from sub-Saharan Africa. Brent et al. (2006) reported a rate of 2% in paediatric outpatients in Kenya [20] and Thriemer et al. (2012) reported a rate of 4% in outpatients of all ages in Zanzibar [21]. Yet, case numbers in our study were too small to assess effects at pathogen level apart from NTS. It remains unclear whether NTS was the main pathogen associated with urbanicity or if contrary effects in the heterogeneous group of pathogens obscured each other.

The observed association between NTS and urbanicity might be affected by associations between NTS and *Plasmodium falciparum* malaria. Several studies reported high NTS incidences in areas with high malaria prevalence and lower NTS numbers in areas with low malaria endemicity [22,23]. On a global scale, urbanization has been shown to coincide with decreased malaria transmission [24,25]. Even though it is unclear, whether these general findings can be applied to the complex epidemiological situation within a transition zone, they still indicate the role of *P*. *falciparum* malaria as a potential confounder for urbanicity. In our study malaria might consequently mediate the effect of urbanicity on NTS. Due to a selection bias (so called Berkson’s bias) we were, however, not able to consider malaria in the regression analyses. In our hospital-based study, both cases and controls were recruited amongst symptomatic (febrile) patients. Hence, recruitment of patients without NTS was dependant on an alternative cause of fever, e.g. *P*. *falciparum* malaria. This biased the distribution of alternative pathogens among cases and controls and hampered further multivariate regression analyses [26,27].

In our study, the proportion of IBI decreased from the lowest to the highest SES group. Data on the relationship between SES and specific infectious diseases are scarce. In most studies, associations with non-specific acute febrile illnesses were reported [28] and these results are heterogeneous. While, for example, in one study conducted in four African countries, Ghana, Nigeria, Kenya and Sierra Leone, a higher likelihood of acute febrile illness in children with low SES was shown [29], studies from Tanzania and Ethiopia did not support this finding [28,30]. Furthermore, the analysis of demographic health surveys from 22 African countries yielded only a weak correlation between individual poverty and non-specific fever. However, there was evidence that the incidence of fever was influenced by the general level of wealth in the community [31]. To disentangle the effects of individual socio-economic and external community factors, we constructed multi-component measures of both determinants. In the multivariate model, effects of SES and urbanicity on bacteraemia were similar to those from the bivariate models. This provides evidence that both factors are considerable independent risk factors for childhood bacteraemia. Urbanicity and SES may influence the risk of bacteraemia via different mechanisms. Some key features of urbanicity are potential protective factors for bacteraemia, namely availability of tap water and sewage systems, access to education and health care, built environment and absence of agriculture [32]. However, low SES might prevent individuals from benefitting from them. Poor education and health related knowledge, low social status, and limited financial means might both increase disease risk, and also delay or prevent healthcare utilisation and the initiation of appropriate treatment measures [28,32,33].

Conventional rural/urban classifications based on surrogate markers, such as population size or density, are inadequate to capture the complex process of urbanization [10]. Especially in transitional societies, where urbanization leads to various forms of settlements, considering the population structure solely will lead to a biased assessment. For example, rapidly growing settlements without proper urban infrastructure would score highly on a population-based scale, but score considerably less on a multi-component scale. Including further urban features, such as public transport, water and sanitation, in this assessment enables the quantification of gradual differences between communities [34]. This allows analyses of health effects on a finer scale as required to differentiate epidemiological outcomes in heterogeneous transition zones.

## Conclusions

The results of this study highlight the importance of individual as well as community factors as independent risk factors for IBI and especially NTS. The processes of urbanization and social transition in sub-Saharan Africa are complex and more data are needed to disentangle their effects on the epidemiology of infectious diseases. Such data will support physicians, public health experts and policy makers to identify disease prevention and treatment needs in the transitional societies of developing countries.

## Supporting Information

S1 Dataset Study data (CSF file). (CSV)
